# Reference datasets of *tuf*A and UPA markers to identify algae in metabarcoding surveys

**DOI:** 10.1016/j.dib.2017.02.013

**Published:** 2017-02-13

**Authors:** Vanessa Rossetto Marcelino, Heroen Verbruggen

**Affiliations:** School of BioSciences, University of Melbourne, Melbourne, Victoria 3010, Australia

**Keywords:** Metabarcoding, *Ostreobium*, *tuf*A, RDP classifier, UPA, Reference sequences

## Abstract

The data presented here are related to the research article “Multi-marker metabarcoding of coral skeletons reveals a rich microbiome and diverse evolutionary origins of endolithic algae” (Marcelino and Verbruggen, 2016) [1]. Here we provide reference datasets of the elongation factor Tu (*tuf*A) and the Universal Plastid Amplicon (UPA) markers in a format that is ready-to-use in the QIIME pipeline (Caporaso et al., 2010) [2]. In addition to sequences previously available in GenBank, we included newly discovered endolithic algae lineages using both amplicon sequencing (Marcelino and Verbruggen, 2016) [1] and chloroplast genome data (Marcelino et al., 2016; Verbruggen et al., in press) [3], [4]. We also provide a script to convert GenBank flatfiles into reference datasets that can be used with other markers. The *tuf*A and UPA reference datasets are made publicly available here to facilitate biodiversity assessments of microalgal communities.

**Specifications Table**

**Table:** 

Subject area	*Biology*
More specific subject area	*Metabarcoding*
Type of data	*Text files (DNA sequence data, metadata and python script)*
How data was acquired	*GenBank data compilation, Amplicon sequencing and Chloroplast genome sequencing*
Data format	*Filtered*
Experimental factors	*Endolithic algae lineages were identified with metabarcoding and chloroplast genome sequencing*
Experimental features	*Genes were extracted from GenBank data, closely related organisms were filtered out and file was converted to a ready-to-use format.*
Data source location	*Melbourne, Australia*
Data accessibility	*The data are available with this article*

**Value of the data**

• The *tuf*A and UPA reference datasets facilitate biodiversity assessments of cyanobacterial and eukaryotic algal communities using high-throughput sequencing.
• When used with the Naive Bayesian Classifier (RDP classifier) implemented in QIIME [2], [5], the taxonomic metadata of the reference datasets provided here allow classifying operational taxonomic units (OTUs) at higher taxonomic ranks when no match is found at lower ranks. For example, an OTU with no close relatives at species or genus level can be classified at the family level, facilitating the interpretation of the results.
• We incorporate in the datasets recently discovered endolithic (limestone-boring) algal lineages [1], [3], [4] to facilitate the identification of these algae in other studies.
• The script provided here facilitates the development of custom reference databases for non-standard metabarcoding markers.

## 1 Data

The datasets of this article provide reference sequences of the elongation factor Tu (*tuf*A) and the Universal Plastid Amplicon (UPA) loci and their corresponding taxonomic information. Supplementary File 1 is a set of identified *tuf*A reference sequences in fasta format. Supplementary File 2 is a tab-delimited file containing the taxonomic information of the *tuf*A reference sequences. The *tuf*A reference dataset contains bacterial and chloroplast *tuf*A sequences, including green algae, red algae, heterokonts, cryptophytes and haptophytes. Supplementary File 3 is a set of identified UPA reference sequences (a fragment of the 23S rDNA) in fasta format. Supplementary File 4 is a tab-delimited file containing the taxonomic information of the UPA reference sequences. This reference dataset contains bacterial and chloroplast 23S rDNA sequences, including cyanobacteria, green algae, red algae, heterokonts, cryptophytes and haptophytes. Supplementary File 5 is a python script that takes a GenBank (.gb) flatfile as input and produces the 2 files needed by the RDP classifier (QIIME version). This script requires Biopython [6].

## 2 Experimental design, materials and methods

We produced reference datasets that can be used with the Naive Bayesian Classifier (RDP classifier) implemented in the QIIME pipeline [2], [5]. Each of these datasets consists of: 1) a fasta file containing the reference DNA sequences and short sequence identifiers and 2) a text file matching the sequence identifiers to their taxonomic metadata. To produce these datasets we first mined sequences from GenBank by querying the marker name and downloading all matching items as full GenBank records. We added endolithic (limestone-boring) green algal lineages discovered with the *tuf*A marker in our study “Multi-marker metabarcoding of coral skeletons reveals a rich microbiome and diverse evolutionary origins of endolithic algae” [1]. We identified these algal lineages in a phylogenetic context [see [1]] and included representatives of the main endolithic clades in the *tuf*A reference dataset. We also retrieved a large diversity of algae with the UPA marker but these lineages did not receive the same nomenclature as the *tuf*A lineages because the correspondence between the *tuf*A and the UPA algal clades was unknown. To solve this issue and match *tuf*A and UPA clades we used chloroplast genome data. The complete chloroplast genomes of two endolithic algal strains – *Ostreobium* HV05042 and SAG699 – were sequenced [3], [4] and added to the UPA reference dataset. Phylogenetically, these strains are in *Ostreobium* Clade 3 and Clade 4, respectively. Since there are no reference sequences for *Ostreobium* Clade 1 and Clade 2 it is possible that OTUs belonging to *Ostreobium* Clades 1 and 2 will be classified as Clades 3 and 4 or will be only classified at higher taxonomic levels.

The reference datasets were equalized so as not to contain identical sequences or a disproportional number of closely related species, which yields downstream benefits for taxonomic assignment [see [7]]. To equalize the datasets and exclude closely related or identical reference sequences, we built a UPGMA tree of the sequences with a JC69 model. We sliced this tree at 0.001 branch length units from the tips, which yielded several clades containing closely related sequences. We kept in the dataset one reference sequence from each of these clades based on their quality (i.e. length and number of undefined bases). For the *tuf*A OTUs obtained in Marcelino and Verbruggen [1] we used a threshold of 0.1 branch length units (1–3 OTUs per family) to not add a disproportionally high amount of endolithic algal lineages in the reference dataset. The reference datasets were converted to a QIIME-friendly format with the gb_2_RDP.py script (Supplementary File 5), which uses the metadata information contained in GenBank files to produce the taxonomic metadata required by RDP. The gb_2_RDP.py script is also available at:

https://github.com/vrmarcelino/Make_Ref_Dataset/blob/master/gb_2_RDP.py

## References

[1] Marcelino V.R., Verbruggen H. (2016). Multi-marker metabarcoding of coral skeletons reveals a rich microbiome and diverse evolutionary origins of endolithic algae. Sci. Rep..

[2] Caporaso J.G., Kuczynski J., Stombaugh J., Bittinger K., Bushman F.D., Costello E.K., Fierer N., Peña A.G., Goodrich J.K., Gordon J.I., Huttley G.A., Kelley S.T., Knights D., Koenig J.E., Ley R.E., Lozupone C.A., McDonald D., Muegge B.D., Pirrung M., Reeder J., Sevinsky J.R., Turnbaugh P.J., Walters W.A., Widmann J., Yatsunenko T., Zaneveld J., Knight R. (2010). QIIME allows analysis of high-throughput community sequencing data. Nat. Methods.

[3] Marcelino V.R., Cremen M.C., Jackson C.J., Larkum A.A., Verbruggen H. (2016). Evolutionary dynamics of chloroplast genomes in low light: a case study of the endolithic green alga *Ostreobium quekettii*. Genome Biol. Evol..

[4] Verbruggen H., Marcelino V.R., Guiry M.D., Cremen M.C.M., Jackson C.J. (2017). Phylogenetic position of the coral symbiont *Ostreobium* (Ulvophyceae) inferred from chloroplast genome data. J. Phycol..

[5] Wang Q., Garrity G.M., Tiedje J.M., Cole J.R. (2007). Naive Bayesian Classifier for rapid assignment of rRNA sequences into the new bacterial taxonomy. Appl. Environ. Microbiol..

[6] Cock P.J.A., Antao T., Chang J.T., Chapman B.A., Cox C.J., Dalke A., Friedberg I., Hamelryck T., Kauff F., Wilczynski B., de Hoon M.J.L. (2009). Biopython: freely available Python tools for computational molecular biology and bioinformatics. Bioinformatics.

[7] Newton I.L., Roeselers G. (2012). The effect of training set on the classification of honey bee gut microbiota using the Naive Bayesian Classifier. BMC Microbiol..

